# Supplementary material for: Genetic determinants of cellular addiction to DNA polymerase theta
Source: Nat Commun. 2019 Sep 19;10:4286. doi: 10.1038/s41467-019-12234-1 (PMC6753077; doi:10.1038/s41467-019-12234-1)
Supplement: Supplementary file 1 — Supplementary Information [file 41467_2019_12234_MOESM1_ESM.pdf]

Supplementary information

Genetic Determinants of Cellular Addiction to DNA Polymerase Theta

Feng et al.

Supplementary Figures

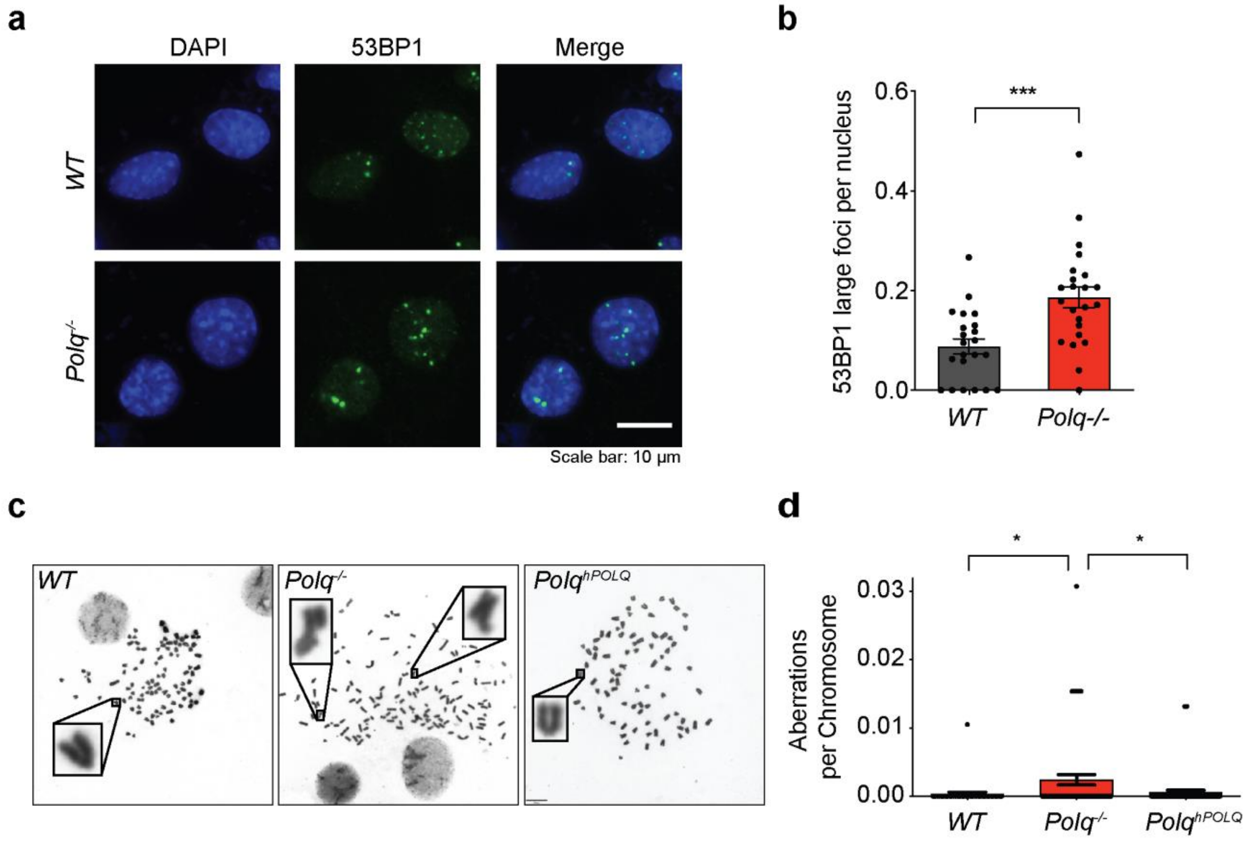

Supplementary Figure 1: *Polq*$^{-/-}$ MEFs exhibit increased levels of spontaneous DSBs and chromosomal aberrations. (a-b) Immunofluorescence (IF) analysis of *WT* and *Polq*$^{-/-}$ cells stained with DAPI and antibodies specific for 53BP1 (n = 3 biologically independent experiments) (a). (b) Quantification of (a). (c-d) Metaphase aberrations are shown in *WT*, *Polq*$^{-/-}$ and *Polq*^{hPOLQ} MEFs (c), scale bar: 10 µm. (d) are quantification of (c). 35 metaphase spreads for each condition were scored. Significance assessed by unpaired, two-tailed t-test. *, p < 0.05 and ***, p<0.001.

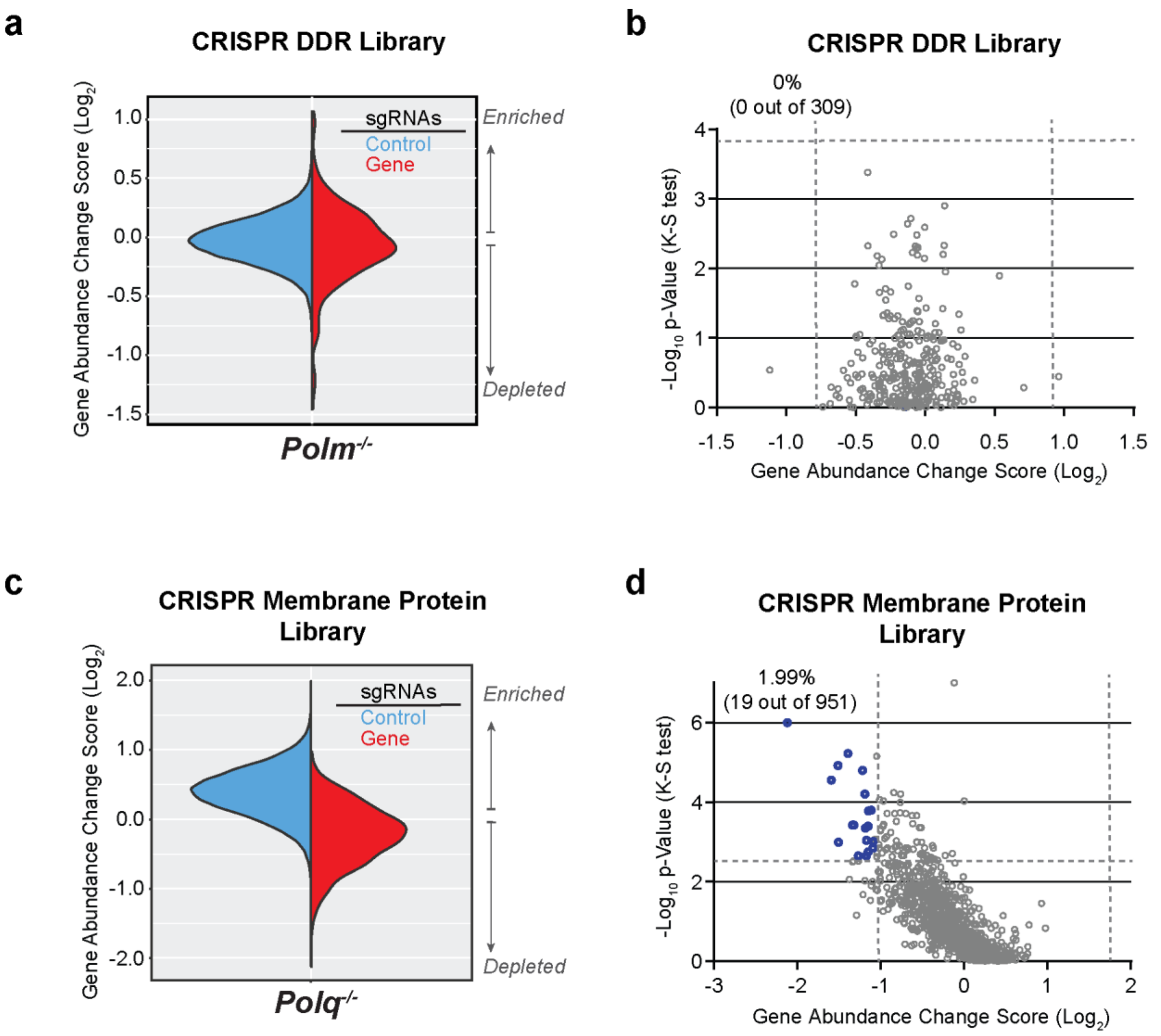

Supplementary Figure 2: Identification of *Polm* synthetic lethal genes by CRISPR screening and Polq synthetic lethal genes by CRISPR membrane protein screening. (a) Violin plot of Gene Abundance Change Scores (Log_2) for DDR gene targeting sgRNAs (red) and non-targeting control sgRNAs (blue) in *Polm*$^{-/-}$ relative to *WT* MEFs. (b) Volcano plot of Gene Abundance Change Scores (*Polm*$^{-/-}$ versus *Polm*$^{-/-}$ *WT*) and $-Log_{10}$ p-value of the Kolmogorov-Smirnov test for DDR gene-targeting sgRNAs relative to non-targeting control sgRNAs. Thresholds for statistical significance are indicated by dashed lines (see Methods for details). Grey dots represent non-significant Gene Abundance Changes Scores. (c) Violin plot of Gene Abundance Change Scores (Log_2) for membrane gene targeting sgRNAs (red) and non-targeting control sgRNAs (blue) in *Polq*$^{-/-}$ relative to *WT* MEFs. (d) Volcano plot of Gene Abundance Change Scores (*Polq*$^{-/-}$ versus *Polq*$^{-/-}$ *WT*) and $-Log_{10}$ p-value of the Kolmogorov-Smirnov test for DDR gene-targeting sgRNAs relative to non-targeting control sgRNAs.

Thresholds for statistical significance are indicated by dashed lines (see Methods for details). Genes with statistically significant (Blue dots) and non-significant (Grey dots) Gene Abundance Changes Scores are indicated.

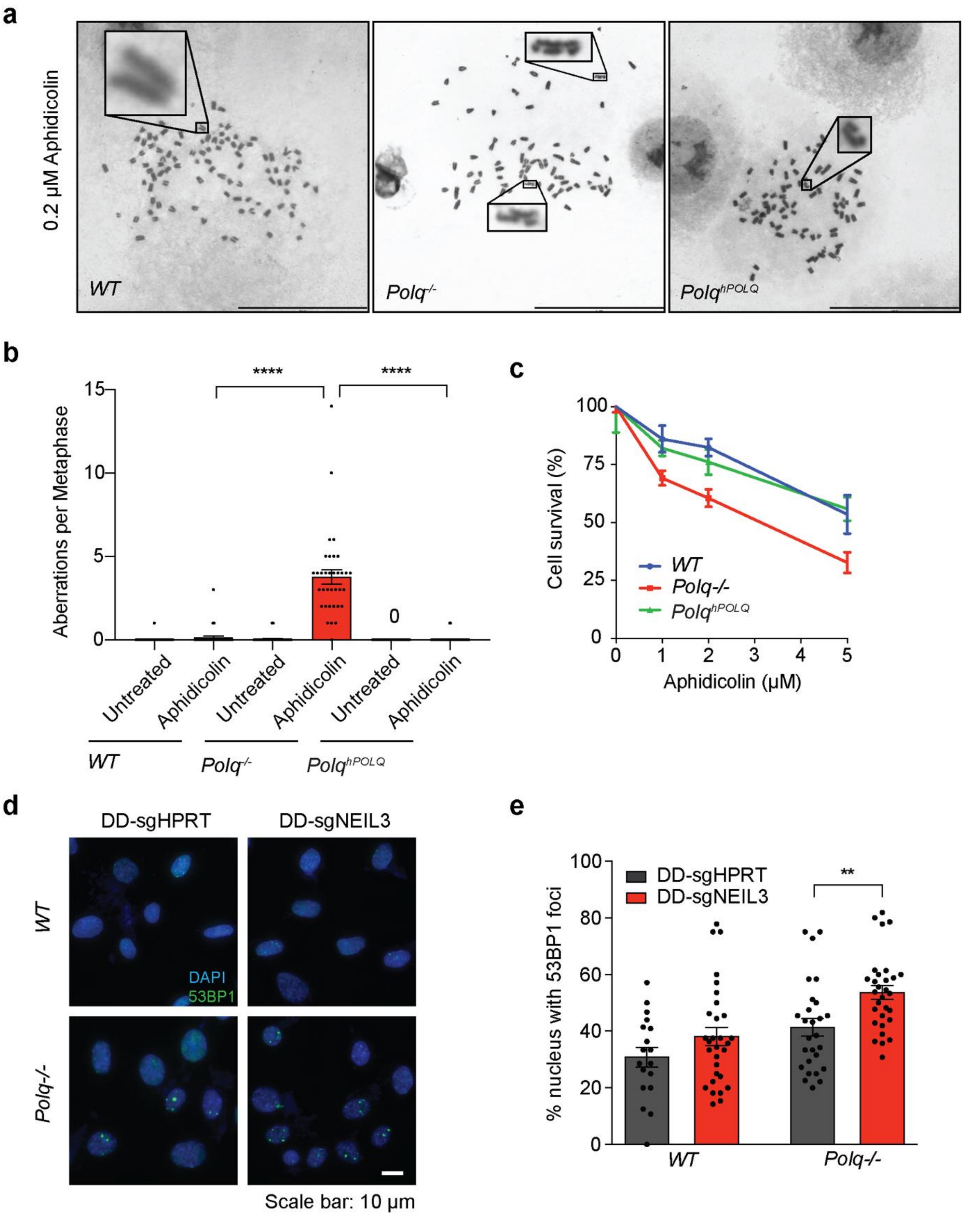

Supplementary Figure 3: *Polq* is required for replication stress associated DNA damage. (a) Metaphase aberrations are shown in *WT*, *Polq*$^{-/-}$ and *Polq*^{hPOLQ} cells 12 hours after treatment with 0.2 μM aphidicolin (APH). Scale bar = 50 μm. (b) Quantification of (a) and shown are

mean± SEM. 35 metaphase spreads for each condition were scored. Significance determined using an unpaired, two-tailed t-test (****, $p<0.0001$). (c) Colony formation assay ± APH, 1, 2, 5 μM in *WT*, *Polq*$^{-/-}$ and *Polq*^{hPOLQ} cells. Colonies were counted from triplicate experiments. Data shown are the mean ± SEM (n = 3). (d-e) Immunofluorescence (IF) analysis of *WT* and *Polq*$^{-/-}$ cells with either sgControl (DD-sgHPRT) or sgNEIL3 (DD-sgNEIL3) stained with DAPI and antibodies specific for Rad51 (n = 3 biologically independent experiments) (d). (e) Quantification of (d). Significance determined using an unpaired, two-tailed t-test (**, $p<0.01$).

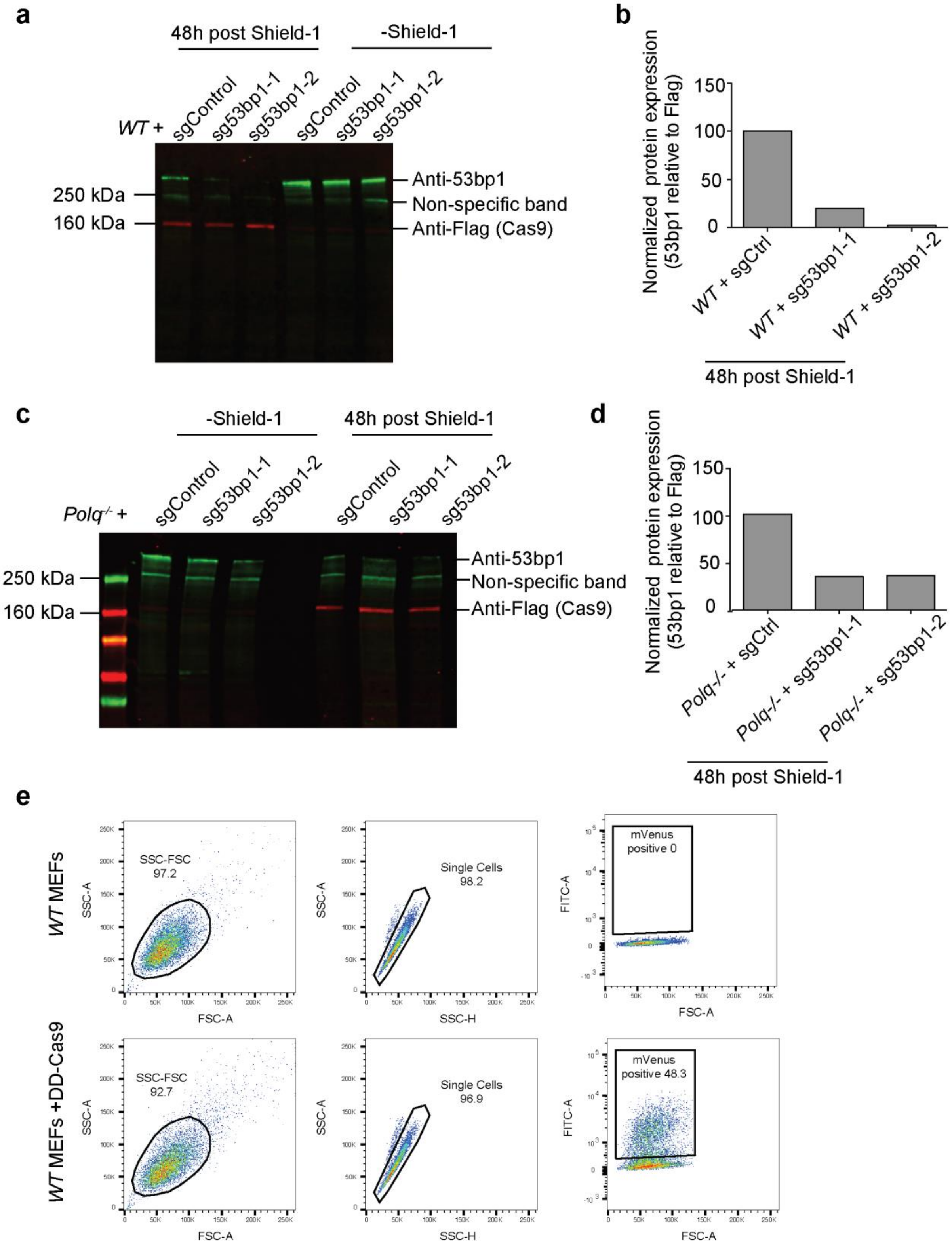

a
48h post Shield-1
-Shield-1
WT +
sgControl
sg53bp1-1
sg53bp1-2
sgControl
sg53bp1-1
sg53bp1-2
250 kDa
160 kDa
Anti-53bp1
Non-specific band
Anti-Flag (Cas9)
b
Normalized protein expression (53bp1 relative to Flag)
150
100
50
0
WT + sgCtrl
WT + sg53bp1-1
WT + sg53bp1-2
48h post Shield-1
c
-Shield-1
48h post Shield-1
Polq-/- +
sgControl
sg53bp1-1
sg53bp1-2
sgControl
sg53bp1-1
sg53bp1-2
250 kDa
160 kDa
Anti-53bp1
Non-specific band
Anti-Flag (Cas9)
d
Normalized protein expression (53bp1 relative to Flag)
150
100
50
0
Polq-/- + sgCtrl
Polq-/- + sg53bp1-1
Polq-/- + sg53bp1-2
48h post Shield-1
e
WT MEFs
SSC-FSC
97.2
Single Cells
98.2
mVenus positive 0
WT MEFs +DD-Cas9
SSC-FSC
92.7
Single Cells
96.9
mVenus positive 48.3
SSC-A
FSC-A
SSC-H
FITC-A

Supplementary Figure 4: Validation of inducible 53bp1 knockdown using DD-Cas9.

Western blot of (a-b) *WT* and (c-d) *Polq*$^{-/-}$ MEFs expressing destabilized Cas9 (DD-Cas9)-sgControl or sg53bp1, with or without treatment with Shield-1 (200ng/ml) for 48 hours. Whole cell lysates were analyzed by Western blot with an anti-53bp1 or anti-Flag antibody, demonstrating Shield1-dependent stabilization of Flag-Cas9 and degradation of 53bp1. (b, d) shows quantification of 53bp1 protein expression, normalized to Flag-Cas9. (e) Gate of cells and percentage of mVenus-positive cells are measured by flow cytometry.

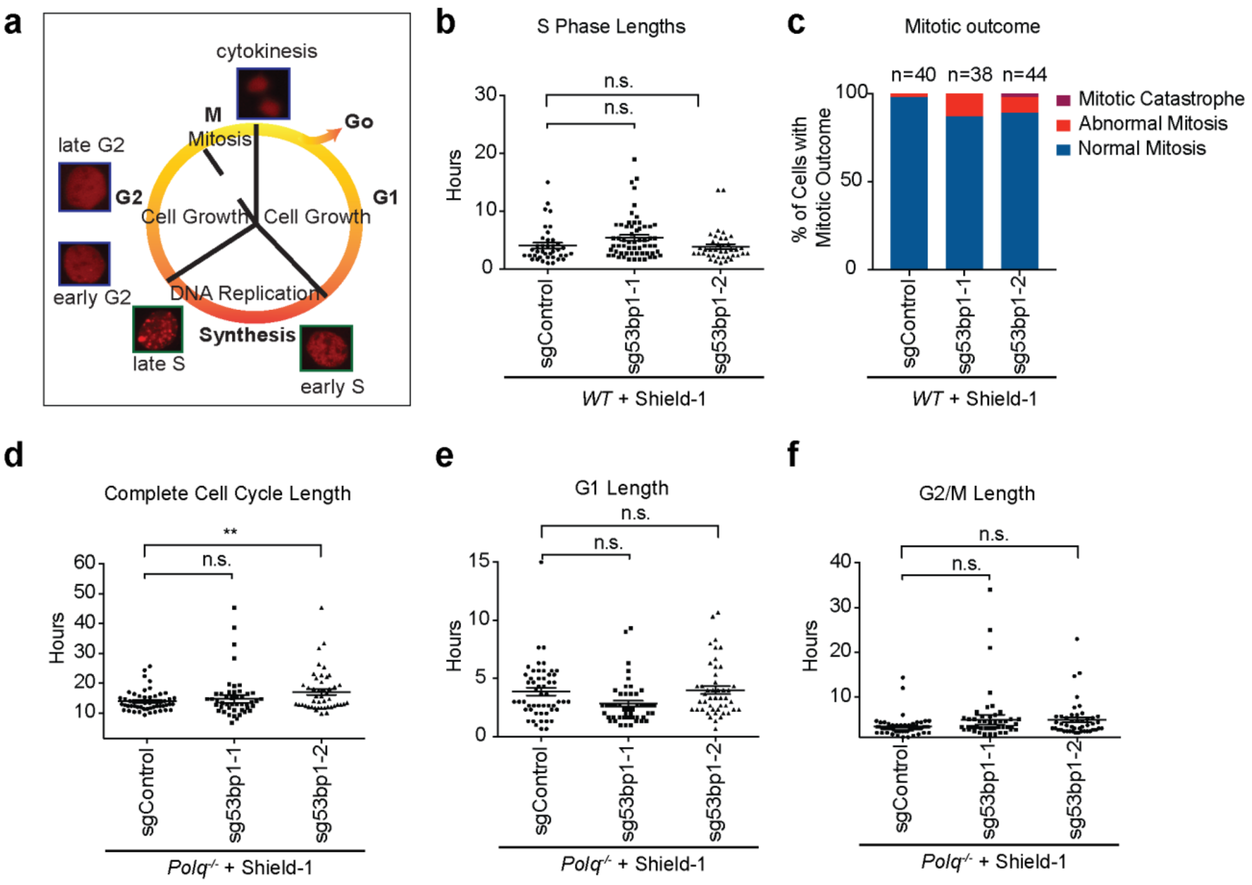

Supplementary Figure 5: Analysis of cell cycle kinetics using a PCNA-mCherry reporter and time-lapse microscopy. (a) Images show typical PCNA-mCherry fluorescence images at the indicated cell cycle phases. Onset of S-phase is recognizable by the development of PCNA-mCherry nuclear foci. (b) Length of S Phase in *WT* + sgCtrl, *WT* + sg53bp1-1, and *WT* + sg53bp1-2 Mouse Embryonic Fibroblasts (MEFs) treated with Shield-1. (c) Mitotic outcome of individual cells was tracked and reported here. (d) Length of complete cell cycle, (e) length of G1 phase and (f) length of G2/M phase in *Polq*$^{-/-}$ + sgCtrl, *Polq*$^{-/-}$ + sg53bp1-1, and *Polq*$^{-/-}$ + sg53bp1-2 MEFs treated with Shield-1. Statistical significance was assessed by two-tailed t-tests. *p < 0.05, **p< 0.01, ***p<0.001 and ****p < 0.0001.

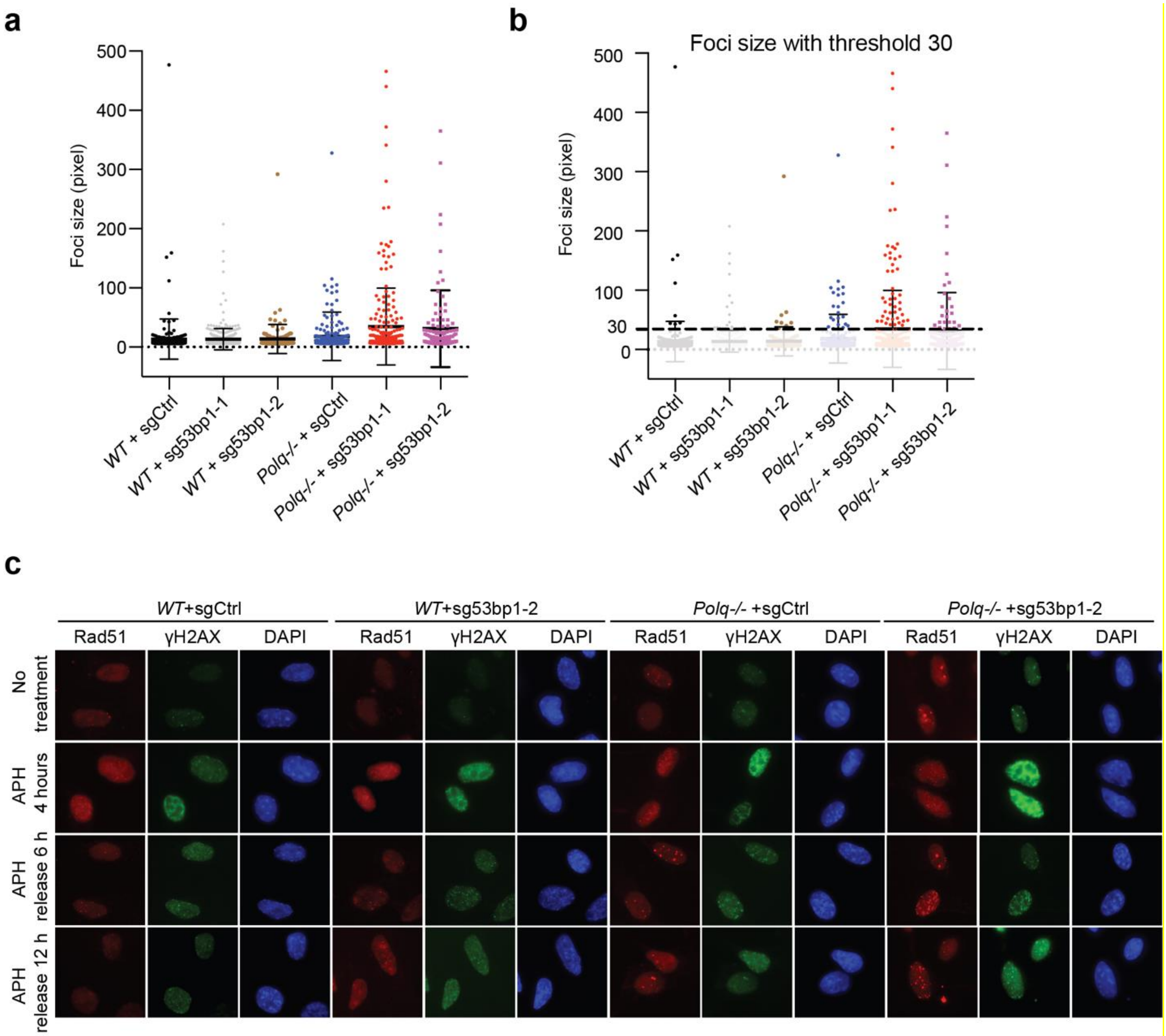

Supplementary Figure 6: Measurement of Rad51 foci size. (a) Distribution of Rad51 foci size is shown for the indicated genotypes. (b) Large foci are defined as those that are larger than pixel area of 30, which eliminates the majority of endogenous foci observed in *WT* cells. A similar size threshold was used for all of the Rad51 foci analyses in this paper. (c) examples of Rad51 and γH2AX IF after treatment and release with 10µM Aphidicolin, in support of Figure 3e.

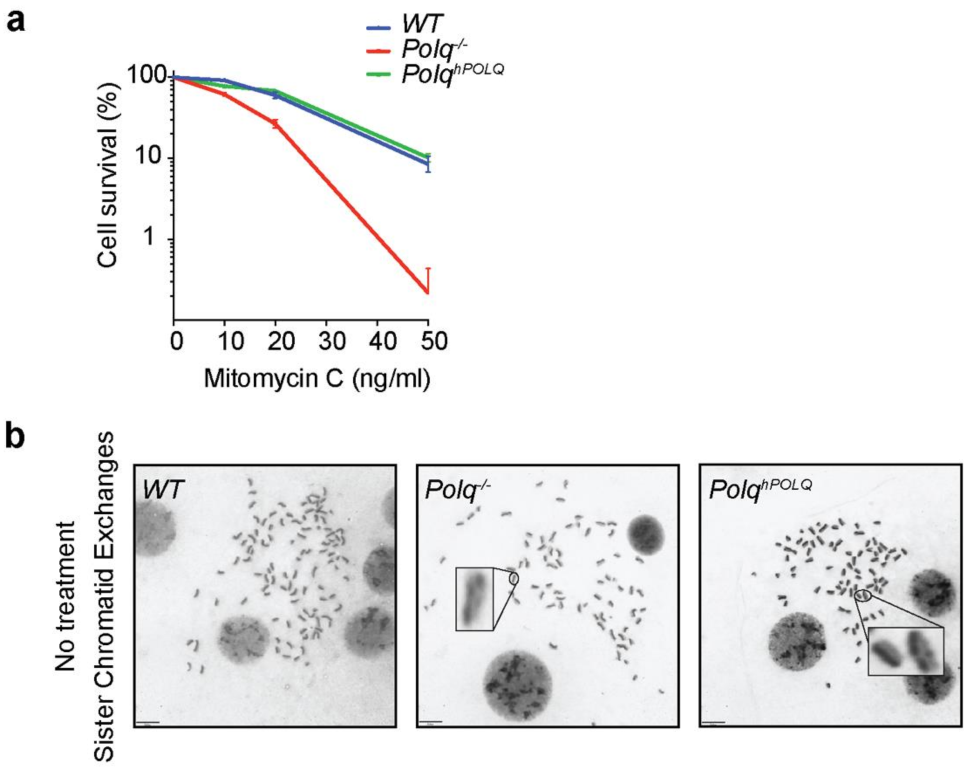

Supplementary Figure 7: (a) Colony formation assay ± MMC, 10, 20, 50 ng/ml in *WT*, *Polq*$^{-/-}$ and *Polq*^{hPOLQ} cells. Colonies were counted from triplicate experiments. Data shown are the mean ± SEM (n = 3). (b) Sister chromatid exchanges are shown in *WT*, *Polq*$^{-/-}$ and *Polq*^{hPOLQ} cells. Scale bar = 10 µm.

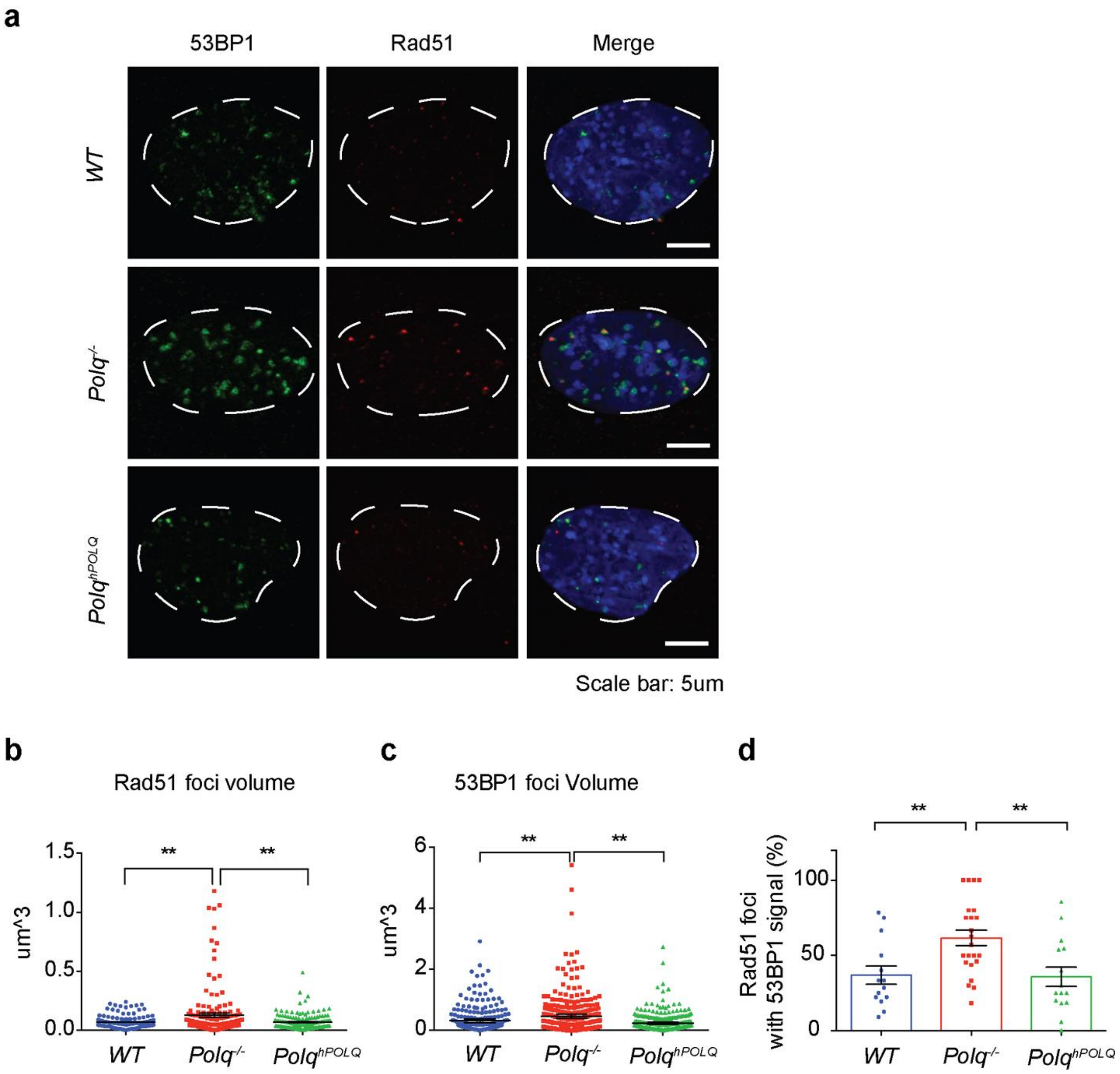

Supplementary Figure 8: Morphology of Rad51 and 53BP1 foci after PDS exposure in *WT* and *Polq*$^{-/-}$ cells. (a-d) IF was performed in *WT*, *Polq*$^{-/-}$ and *Polq*^{hPOLQ} cells 6 hours after treatment with 5 µM PDS. Cells were stained with DAPI and antibodies specific for Rad51 and 53BP1 (n = 3 biologically independent experiments). Image acquisition was performed using the LSM 880 Airyscan. Volume of (b) Rad51 and (c) 53BP1 foci are measured by Imaris image analysis software. (d) Percentage of Rad51 foci with co-localization of 53BP1 in the noted cell lines. Statistical significance was assessed by two-tailed t-tests (**, $p < 0.01$).

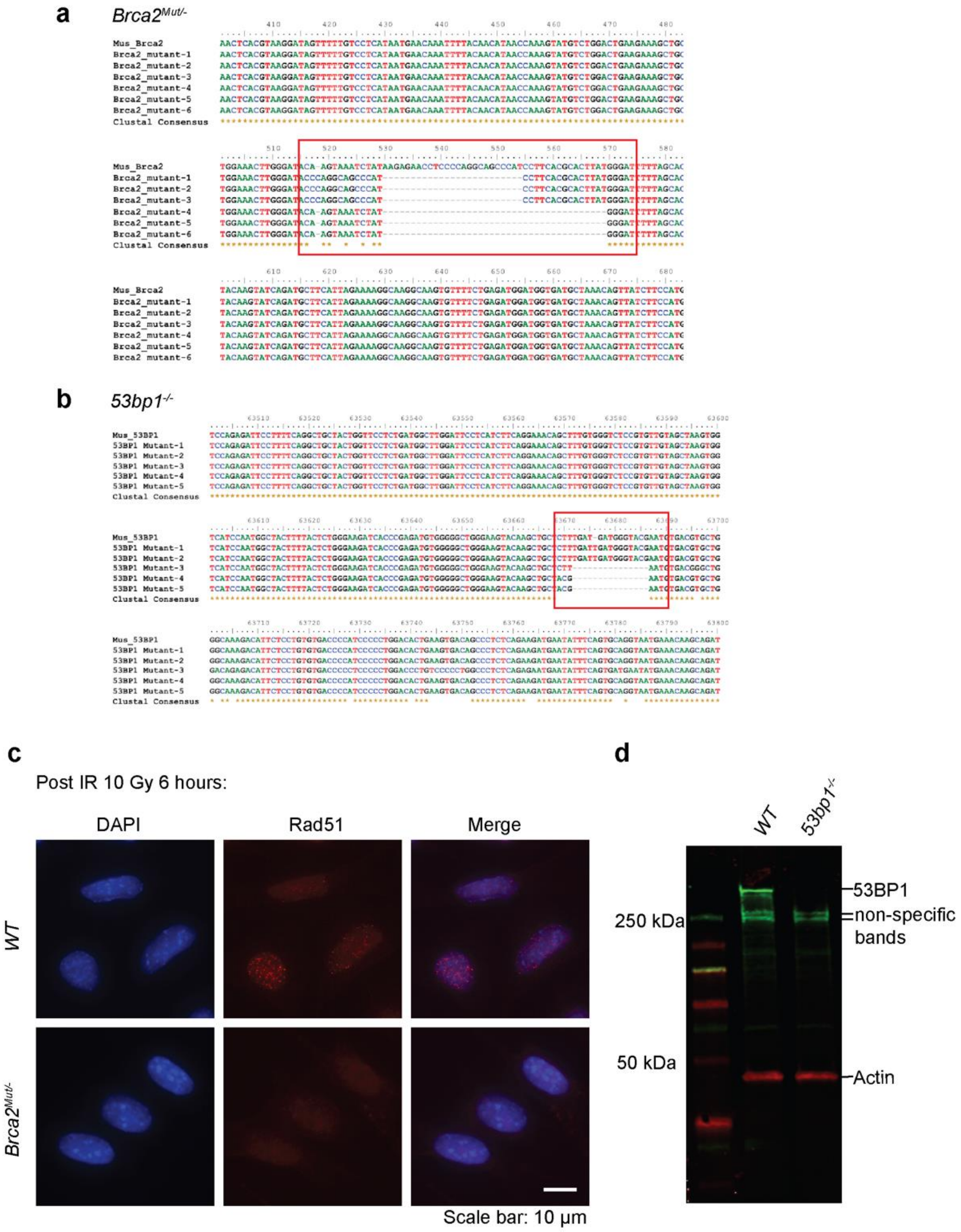

Supplementary Figure 9: Validation of *Brca2*$^{Mut/-}$ and *53bp1*$^{-/-}$ MEFs generated by Alt-R CRISPR gene editing. (a-b) Sanger sequencing analysis of CRISPR edited locus in (a) *Brca2*$^{Mut/-}$ and (b) *53bp1*$^{-/-}$ MEF clones**.** The locus of interest was PCR-amplified and cloned into a TOPO vector for sequencing analyses. Each line of sequence shown was derived from a different TOPO clone and aligned to show differences. The *53bp1*$^{-/-}$ MEF clone has one allele

with a 1bp insertion, and another allele with a 14bp deletion—both resulting in frameshift mutations. The *Brca2*$^{Mut/-}$ MEF clone has one allele with a 40bp deletion, causing a frameshift mutation, whereas the other *Brca2* allele has a 25bp deletion and a 1bp insertion, resulting in an in-frame deletion with 4 mutant amino acids and 8 deleted amino acids. (c) IF analysis of *WT* and *Brca2*$^{Mut/-}$ MEFs stained with DAPI and Rad51 6 hours after exposure to 10Gy ionizing radiation. Rad51 foci were significantly decreased in *Brca2*$^{Mut/-}$ MEFs, indicative of functional deficiency in HR. (d) Western blot of *WT* and *53bp1*$^{-/-}$ MEFs. 53BP1 was detected in *WT* cells but not in *53bp1*$^{-/-}$ cells. Actin was used for a loading control.

Supplementary Note 1

(1) Normalization of library counts.

$$TC_{Norm} = \frac{sgCount}{\sum sgCount}$$

(2) Combine Normalized Counts from Replicates.

$$gTC_{Norm} = \left(\prod_{k=1}^{n} \mathrm{TCnorm}_k\right)^{1/n}$$

(3) Normalize to Plasmid Sample and Mask any sgGuide's Missing from the Plasmid Sample. Determine the sgGuide Abundance Change Score for random sampling test.

$$TCD_{Norm} = \frac{Experimental\ gTC_{Norm}}{Plasmid\ gTC_{Norm}}$$

$$sgGuideABC = log_2(Experimental\,TCDnorm - Control\,TCDnorm)$$

(4) Gene Abundance Change Scores.

$$geneABC = log_2\left(\left(\prod_{k=1}^{n} \mathrm{Experimental\ TCDnorm}_k - \mathrm{Control\ TCDnorm}_k\right)^{1/n}\right)$$

sgCount: Count of individual sgGuide sequence in a single sequencing library.
TC_{Norm}: sgCount normalized to total counts in single sequencing library.
gTC_{Norm}: Geometric mean of the TC_{Norm} values from the replicate libraries.
TD_{Norm}: gTC_{Norm} data normalized to the Plasmid sample. This is the plasmid library used to produce the lentiviaral library for all experiments. Represents the starting diversity of the library.
sgGuideABC: sgGuide Abundance Change Scores.
geneABC: Gene Abundance Change Scores.
