# Supplementary material for: Genetic determinants of cellular addiction to DNA polymerase theta
Source: Nat Commun. 2019 Sep 19;10:4286. doi: 10.1038/s41467-019-12234-1 (PMC6753077; doi:10.1038/s41467-019-12234-1)
Supplement: Supplementary file 3 — Description of Additional Supplementary Files [file 41467_2019_12234_MOESM3_ESM.pdf]

Description of Additional Supplementary Files
Supplementary Data 1:

Sequence of oligonucleotides used to clone DDR-CRISPR library.

Supplementary Data 2:

Results of DDR-CRISPR screens in *WT*, *Polq*$^{-/-}$ and *Polq*^{hPOLQ} cells by our Völundr. Tab 1 named Gene List: Gene Abundance Change Scores and Kolmogorov-Smirnov p-values. Significant genes are listed in blue, non-significant genes are listed in grey. Tab 2 named Gene Categories: Category membership of genes. Used to generate figure 1e.

Supplementary Data 3:

Tab 1 named DDR-Polm: Results of DDR-CRISPR screens in *WT* and *Polm*$^{-/-}$ cells using the Völundr pipeline. Tab 2 named Membrane-Polq: Results of membrane-CRISPR screens in *WT* and *Polq*$^{-/-}$ cells using Völundr. Significant genes are listed in blue, non-significant genes are listed in grey.

Supplementary Data 4:

TCGA human data, named tcga_brca_polqSL_mutations: list of PolqSL gene mutation in each TCGA sample.

Supplementary Data 5:

TCGA human data, named tcga_brca_mhd_cosmic3: List of MHD, COSMIC3 score, and PolqSL mutation status for each TCGA sample.

Supplementary Data 6:

TCGA human data, named tcga_brca_mhd_wgs_wes: Same list as above but for the TCGA samples that also had wgs done with MHD_WGS scores

Supplementary Data 7:

Oligonucleotide Sequences, Probe Sequences and Miscellaneous sgGuide sequences used in the study.
