# Supplementary material for: Genetic determinants of cellular addiction to DNA polymerase theta
Source: Nat Commun. 2019 Sep 19;10:4286. doi: 10.1038/s41467-019-12234-1 (PMC6753077; doi:10.1038/s41467-019-12234-1)
Supplement: Supplementary file 11 — Reporting Summary [file 41467_2019_12234_MOESM11_ESM.pdf]

nature research

Corresponding author(s): Gaorav P. Gupta, MD PhD, gaorav@med.unc.edu

Last updated by author(s): YYYY-MM-DD

Reporting Summary

Nature Research wishes to improve the reproducibility of the work that we publish. This form provides structure for consistency and transparency in reporting. For further information on Nature Research policies, see Authors & Referees and the Editorial Policy Checklist.

Statistics

For all statistical analyses, confirm that the following items are present in the figure legend, table legend, main text, or Methods section.

n/a	Confirmed	
☐	☒	The exact sample size (*n*) for each experimental group/condition, given as a discrete number and unit of measurement
☒	☐	A statement on whether measurements were taken from distinct samples or whether the same sample was measured repeatedly
☐	☒	The statistical test(s) used AND whether they are one- or two-sided<br>*Only common tests should be described solely by name; describe more complex techniques in the Methods section.*
☒	☐	A description of all covariates tested
☐	☒	A description of any assumptions or corrections, such as tests of normality and adjustment for multiple comparisons
☐	☒	A full description of the statistical parameters including central tendency (e.g. means) or other basic estimates (e.g. regression coefficient) AND variation (e.g. standard deviation) or associated estimates of uncertainty (e.g. confidence intervals)
☐	☒	For null hypothesis testing, the test statistic (e.g. *F*, *t*, *r*) with confidence intervals, effect sizes, degrees of freedom and *P* value noted<br>*Give P values as exact values whenever suitable.*
☒	☐	For Bayesian analysis, information on the choice of priors and Markov chain Monte Carlo settings
☒	☐	For hierarchical and complex designs, identification of the appropriate level for tests and full reporting of outcomes
☒	☐	Estimates of effect sizes (e.g. Cohen's *d*, Pearson's *r*), indicating how they were calculated

Our web collection on statistics for biologists contains articles on many of the points above.

Software and code

Policy information about availability of computer code

Data collection	Raw Ion sequencing output was converted to FASTQ format using Ion software.
Data analysis	Code for Völundr v1.0.0 has been made publicly available at https://github.com/pkMyt1/Volundr. Graphpad Prism v8. Analysis of TCGA data using ABRA2, biobambam2, Strelka, UNCeqR, Cadabra, Ensembl Variant Effect Predictor, SeqPurge, Star, Salmon. R Studio v1.2.1335, Fiji v1.5.1s, Quantsoft Analysis Pro v1.0

For manuscripts utilizing custom algorithms or software that are central to the research but not yet described in published literature, software must be made available to editors/reviewers. We strongly encourage code deposition in a community repository (e.g. GitHub). See the Nature Research guidelines for submitting code & software for further information.

Data

Policy information about availability of data

All manuscripts must include a data availability statement. This statement should provide the following information, where applicable:

- Accession codes, unique identifiers, or web links for publicly available datasets
- A list of figures that have associated raw data
- A description of any restrictions on data availability

Sequencing data is available at [https://www.ncbi.nlm.nih.gov/sra/PRJNA556352]. All unanalyzed raw data is available at [https://figshare.com/projects/Genetic_Determinants_of_Cellular_Addiction_to_DNA_Polymerase_Theta/67331]. For live cell imaging data, due to their large size, please request them from our corresponding author. The source data underlying Figs 1d, 2b, 2d, 2f-g, 3b, 3d-e, 4b-c, 4e-f, 5a-b, 5d-e, 6b-e and Supplementary Figs 1, 3, 5, 6, 7a and 8 are provided as a source data file.

Field-specific reporting

Please select the one below that is the best fit for your research. If you are not sure, read the appropriate sections before making your selection.

☒ Life sciences ☐ Behavioural & social sciences ☐ Ecological, evolutionary & environmental sciences

For a reference copy of the document with all sections, see nature.com/documents/nr-reporting-summary-flat.pdf

Life sciences study design

All studies must disclose on these points even when the disclosure is negative.

Sample size	No sample size calculation was performed. At least 3 technical and/or biological replicates for each sample was done.
Data exclusions	No data were excluded from analysis or reporting
Replication	All experiments were reliably reproduced as stated in the text.
Randomization	Not applicable as human or animal subjects were not used in this study.
Blinding	Not applicable as human or animal subjects were not used in this study.

Reporting for specific materials, systems and methods

We require information from authors about some types of materials, experimental systems and methods used in many studies. Here, indicate whether each material, system or method listed is relevant to your study. If you are not sure if a list item applies to your research, read the appropriate section before selecting a response.

Materials & experimental systems

n/a	Involved in the study	
☐	☒	Antibodies
☐	☒	Eukaryotic cell lines
☒	☐	Palaeontology
☒	☐	Animals and other organisms
☒	☐	Human research participants
☒	☐	Clinical data

Methods

n/a	Involved in the study	
☒	☐	ChIP-seq
☐	☒	Flow cytometry
☒	☐	MRI-based neuroimaging

Antibodies

Antibodies used

Antibody# Species# Company Cat# Application Dilution
Rad51 Mouse Novus Biologicals NB100-148, IF 1:500
gamma H2AX Rabbit Trevigen 4418-APC-100, IF 1:500
53BP1 Rabbit Bethyl A300-272A, IF 1:500 WB 1:5000
Flag Mouse Sigma-Aldrich F1804-200UG, WB 1:5000
Actin Mouse Sigma-Aldrich A1978-100UL, WB 1:10,000
DAPI ThermoFisher D1306, IF 1:10,000
Rhodamine Goat Anti-Mouse IgG (H+L) Jackson ImmunoResearch 115-025-146, IF 1:500
FITC Goat Anti Rabbit IgG (H+L) Jackson ImmunoResearch 111-095-144, IF 1:500
IRDye® 800CW Goat anti-Rabbit IgG (H + L) LI-COR Biosciences P/N 926-32211, WB 1:10,000
IRDye® 680RD Goat anti-Mouse IgG (H + L) LI-COR Biosciences P/N 926-68070, WB 1:10,000

Validation

Appropriate positive and negative controls were included in the experimental design to confirm the antibodies were specific.

Eukaryotic cell lines

Policy information about cell lines

Cell line source(s)	WT MEFs; Polq-/- MEFs; Polq-/- + POLQ; HEK293T; PolM-/-; PolM+/+; As cited in methods.
Authentication	Loss of Polq function was determined as described in the methods using functional assays. No further authentication of the cell lines was done.
Mycoplasma contamination	All cells are routinely tested for and found to be mycoplasma free as described in the Methods.

Commonly misidentified lines (See ICLAC register)	No commonly misidentified cell lines were used.

Flow Cytometry

Plots

Confirm that:

☒ The axis labels state the marker and fluorochrome used (e.g. CD4-FITC).

☒ The axis scales are clearly visible. Include numbers along axes only for bottom left plot of group (a 'group' is an analysis of identical markers).

☒ All plots are contour plots with outliers or pseudocolor plots.

☒ A numerical value for number of cells or percentage (with statistics) is provided.

Methodology

Sample preparation	MEFs were trypsinized and fixed in 1% paraformaldehyde in PBS at the time point indicated in the text.
Instrument	The Becton Dickinson LSR II
Software	The data was collected using FACSDiva 8.0.1 and analysed using FlowJo
Cell population abundance	Cells were analyzed by Western Blot as described in the text.
Gating strategy	FSC/SSC gates define single cell population. For each condition, mVenus gates were defined using WT MEFs cell population as a negative control.

☒ Tick this box to confirm that a figure exemplifying the gating strategy is provided in the Supplementary Information.
